# Neobaicalein, a flavonoid from the *Scutellaria litwinowii* Bornm. & Sint. ex Bornm. induced apoptosis in human leukemic cell lines

**DOI:** 10.22038/IJBMS.2023.66240.14616

**Published:** 2023-03

**Authors:** Seyed Ahmad Emami, Elham Ramazani, Seyed Hadi Mousavi, Nasser Vahdati-Mashhadian, Javad Asili, Heydar Parsaee, Zahra Tayarani-Najaran

**Affiliations:** 1 Department of Traditional Pharmacy, School of Pharmacy, Mashhad University of Medical Sciences, Mashhad, Iran; 2 Department of Biology, Faculty of Science, Ferdowsi University of Mashhad, Mashhad, Iran; 3 Medical Toxicology Research Center, Mashhad University of Medical Sciences, Mashhad, Iran; 4 Department of Pharmacognosy, School of Pharmacy, Mashhad University of Medical Sciences, Mashhad, Iran; 5 Department of Pharmacology and Pharmacological Research Center of Medicinal Plants, School of Medicine, Mashhad University of Medical Sciences, Mashhad, Iran; 6 Targeted Drug Delivery Research Center, Pharmaceutical Technology Institute, Mashhad University of Medical Sciences, Mashhad, Iran

**Keywords:** Apoptosis, Caspase, Neobaicalein, Poly (ADP-ribose) – polymerase, *Scutellaria litwinowii*

## Abstract

**Objective(s):**

Neobaicalein is one of the rich plant flavonoids isolated from the roots of *Scutellaria* spp. In this study, we evaluated and compared cytotoxic activity and the related apoptosis mechanisms of neobaicalein from *Scutellaria litwinowii* Bornm. & Sint. ex Bornm on apoptosis-proficient HL-60 cells and apoptosis-resistant K562 cells.

**Materials and Methods:**

Cell viability, cell apoptosis, caspase activity, and apoptosis-related protein expression were measured using MTS assay, propidium iodide (PI) staining and flow cytometry, caspase activity assay, and western blot analysis, respectively.

**Results:**

Neobaicalein significantly reduced cell viability in a dose-dependent manner using the MTS assay (*P*<0.05). The IC_50 _values (µM) against HL-60 and K562 cells after 48 hr treatment were 40.5 and 84.8, respectively. Incubation of HL-60 and K562 cells with 25, 50, and 100 µM neobaicalein for 48 hr, significantly increased the number of apoptotic cells and showed cytotoxic effects compared with the control group. Treatment with neobaicalein significantly increased Fas (*P*<0.05) and the cleaved form of PARP (*P*<0.05), and decreased the Bcl-2 levels (*P*<0.05) in HL-60 cells, whereas neobaicalein significantly increased Bax (*P*<0.05) and the cleaved form of PARP (*P*<0.05), and the caspases of the extrinsic and intrinsic pathways including caspases-8 (*P*<0.0001), -9 (*P*<0.01), and effector caspase-3 (*P*<0.0001) levels in K562 cells compared with the control group.

**Conclusion:**

It seems neobaicalein might cause cytotoxicity and cell apoptosis through interaction with the different apoptosis-related proteins of apoptotic pathways in HL-60 and K562 cells. Neobaicalein may exert a beneficial protective effect in slowing the progression of hematological malignancies.

## Introduction

Flavones, isoflavones, and flavanones are categorized as flavonoids and share a polyphenol structure. Edible plants are considered a source of flavonoids, which are thought to have a preventive role for many diseases including cancer (1). Flavonoids have been implicated as a preventive medication due to their approved anti-oxidant, anti-inflammatory, cardioprotective, and anticarcinogenic activities. Anticancer properties of flavonoids have been emphasized because of several reports on the cytotoxic actions on human and animal tumor cells, and multiple studies on the anti-tumor effects of flavonoids. Considering the action on numerous tumor arrest cascades, including alteration in apoptosis, cell cycle, and angiogenesis signaling pathways, flavonoids are deliberated as promising anticancer agents (1-3). In view of the appropriate relationship between dietary flavonoids and cancer inhibition, these phytochemicals encourage investigators to further study the various underlying mechanisms of potential anticancer activities of plant flavonoids. Currently, the antitumor activity of flavonoids has been investigated and many of them were entered into clinical trials (2, 3).

The genus *Scutellaria* is composed of multipurpose herbs from different species used in traditional Chinese medicine to treat various conditions such as fever, cough, inflammation, dysentery, jaundice, and hypertension (4). Plants related to the genus *Scutellaria* are famed to contain large quantities of flavonoids (5). Baicalein, baicalin, wogonin, and oroxylin A are among the main constituents of flavonoids in *Scutellariae radix *which are well-known for their biological impacts (6). Neobaicalein (skullcapflavone or 5,2’-dihydroxy-6, 7,8,6’-tetramethoxyflavone), a flavonoid of *Scutellaria* *baicalensis* root has been reported to show different activities. Neobaicalein has anti-arthritic and anti-inflammatory activities which are partly due to the inhibition of enzymes responsible for the metabolism of arachidonate in rat leukocytes specifically those that lead to the production of inflammatory products (7). Also, neobaicalein could inhibit the plasminogen activator inhibitor-1 increase induced by trypsin (8). In scheduled screening tests, neobaicalein, as the active constituent of *S. baicalensis* showed a cytotoxic effect on L1210 cells (9). Comparative investigation of the flavones of skullcap, including baicalin, baicalein, wogonin, and neobaicalein revealed the potent cytotoxic activity of neobaicalein (9,10).

In an attempt to investigate the bioactive compounds associated with the cytotoxic properties of *Scutellaria litwinowii* (11), neobaicalein and wogonin were isolated from this species. In spite of numerous studies conducted on the apoptotic effects of oroxylin A, wogonin, baicalein, and baicalin, the chief ingredients in *S.* *radix*, the apoptotic effect of neobaicalein as an important flavonoid found in various species of *Scutellaria *has not yet been confirmed. 

Apoptosis, known as programmed cell death, has a crucial role in various physiological processes (12). Apoptosis is now thought to occur through both mitochondrial (or intrinsic) and death-receptor (or extrinsic) pathways. An increase in mitochondrial permeability of cytochrome c triggers a caspase cascade in the mitochondria-related pathway. Caspase-8 recruited the death-receptor complexes to launch the receptor-mediated pathway of apoptosis (13). Both the intrinsic and extrinsic pathways refereed apoptosis via the caspase-8-mediated cleavage of Bid (14).

In this study, the apoptotic cell death induction of neobaicalein, a flavonoid from the CH_2_Cl_2_ fraction of *S. litwinowi* root extract was investigated in human leukemia cells regarding both mitochondrial and death-receptor pathways. As far as we know, the evaluation of apoptotic induction by neobaicalein has not been evaluated thus far in any other study. The findings of this study encourage the development of a new and less toxic cancer chemotherapeutic agent in the class of plant flavonoids.

## Materials and Methods

***Plant material***

The roots of *S. litwinowii* were collected from Hosseinabad valley in Pivejan (a village 65 km southwest of Mashhad, Razavi Khorasan province, northeast of Iran) and identified by MR Joharchi, from Ferdowsi University of Mashhad Herbarium. A voucher specimen (No. 14175) was deposited in the herbarium of the Faculty of Pharmacy, Mashhad University of Medical Sciences, Mashhad, Iran (15, 16).

***Extraction, isolation, and purification of neobaicalein***

**Figure 1:**
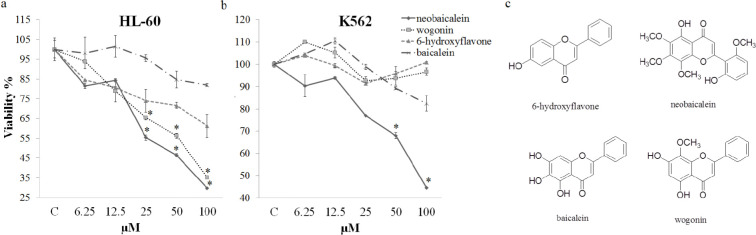
Cell viability: (a) Effects of various concentrations (6.25–100 μM) of neobaicalein, wogonin, 6-hydroxyflavone, and baicalein on the viability of HL-60 cells, (b) Effects of various concentrations (6.25–100 μM) of neobaicalein, wogonin, 6-hydroxyflavone, and baicalein on the viability of K562 cells. (c) Structures of the four compounds. Cytotoxicity was determined using an MTS assay. Values are the mean±SEM of three independent experiments in triplicate. *P<0.05, ** *P*<0.01, ***P<0.001 compared with the control group

In view of our previous work which confirmed the cytotoxic activity of the dichloromethane (CH_2_Cl_2_) extract from the root of *S. litwinowii* on AGS, HeLa, MCF-7, PC12, HL-60, and K562 cells (11, 15, 16), Neobaicalein (5,2’-dihydroxy-6, 7,8,6’- tetramethoxyflavone, Figure 1c) was obtained from the CH_2_Cl_2_ extract over a silica gel column chromatography fractionation followed by purification using reversed-phase semiparaperative high-performance liquid chromatography (HPLC). The purification was carried out on a Wellchrom Knauer system (Herbert Knauer GmbH, Berlin, Germany) that consisted of a Knauer K-1001 pump, using RP18 (250 mm×16 mm, 5 µm) column, eluted isocratically with MeOH/H_2_O (9:1) mixed with 0.05% phosphoric acid v/v (adjusted by triethylamine to pH 3) at 2 ml/min. The UV-Vis detector was set at 270 nm (15). The identification was confirmed by comparison of ^13^C-NMR, ^1^H-NMR, and HMBC spectra (Table 1), and melting points of the parent compound with the reported data (17, 18). 

***Cell cultures and treatment agents***

HL-60 (code number: C217) and K562 (code number: C122) cells were purchased from Cell Bank at the Pasteur Institute (Tehran, Iran) and were cultured in an RPMI-1640 medium supplemented with 10% fetal bovine serum, 100 U/ml penicillin, and 100 mg/ml streptomycin. Cells were kept at 37 °C in a humidified atmosphere (90%) containing 5% CO_2_. For each concentration and time course study, there was a control sample, which remained untreated and received an equal volume of the culture medium (19). 

For cell viability, HL-60 and K562 cells were treated with 6.25-100 μM of neobaicalein. For flow cytometry analysis, HL-60 and K562 cells were incubated with 25, 50, and 100 μM of neobaicalein. For caspase activity analysis, K562 cells were incubated with 25 and 50 μM of neobaicalein. For western blotting analysis, HL-60 and K562 cells were treated with 6.25-50 μM and 12.5-100 μM of neobaicalein, respectively. 

***Cell proliferation and viability assays***

Reduction of the MTS assay by mitochondrial dehydrogenase in metabolically active cells to formazan is the basis of the MTS assay, which is water-soluble and absorbs at 490 nm (20). For indicating cell viability, HL-60 and K562 cells (4×10^3^ cells per well) were seeded in 96-well plates. Cells were treated as described previously. After 48 hr incubation, CellTiter 96® Aqueous One Solution Reagent (Promega, Madison, WI, USA) was added to each well and incubated for 3 hr. Cell viability was assessed at the absorbance of 600 nm by an ELISA microplate reader and compared with the control group (Awareness, Palm City, FL, USA) (21).

***PI staining***

Detecting of the apoptotic cells was performed after flow cytometry and PI (a quantitative DNA-binding dye) staining of treated cells to identify DNA fragmentation (so-called sub-G1 peaks) (22). Briefly, HL-60 and K562 cells (2×10^5^ cells per well) were cultured in 12-well plates. Cells were treated as described previously for 48 hr. For the flow cytometric analysis, cells were washed with phosphate-buffer saline (PBS). After trypsinization, the cells were harvested and incubated at 4 °C in the dark with 400 μl of hypotonic buffer (50 μg/ml PI in 0.1% sodium citrate and 0.1% Triton X-100) for 30 min before flow cytometry analysis (BD Biosciences, CA, USA) (23,24).

***Caspase activity assay***

Using a caspase colorimetric protease kit the activity of caspases -3, -8, and -9 was quantified. For caspase activity K562 cells (4×10^3^ cells per well) were seeded in 96-well plates and were treated as described previously for 48 hr. The cell was lysed according to the manufacturer’s instruction, and samples with equal protein content were prepared. Then, the cell lysate containing 75 mg of protein with 4 ml of 4 mM pNA-conjugated substrates (DEVD-pNA, IETD-pNA, and LEHD-pNA; substrates for caspases-3, -8, and -9, respectively) was incubated for 3.5 hr at 37 °C. The amount of pNA released was measured and compared with the related control after assessment at 405 nm with the ELISA microplate reader (Awareness, Palm City, FL, USA) (21).

***Western blotting analysis***

According to the protocol of the previous studies (25-27), western blot analysis was used to detect the expression of poly (ADP-ribose) polymerase (PARP), Fas, Bcl-2 associated X- protein (Bax), b-cell lymphoma 2 (Bcl-2), and caspase-3. HL-60 and K562 cells (10^6^ cells/T25 flask) were grown and treated as described previously. Followed by 48 hr of incubation, cells were washed with cool PBS and harvested. Finally, the expression levels of these proteins were normalized with respect to β-actin (Gel Doc UV Alliance, Alliance 4.7, UK). 

***Statistical analysis***

The one-way analysis of variance (ANOVA) followed by the Tukey-Kramer *post hoc* test was used to evaluate the differences between the groups and two-way ANOVA for comparing differences between neobaicalein, wogonin, 6-hydroxyflavone, and baicalein. All results were presented as mean±SEM from triplicate experiments performed in a parallel manner unless otherwise indicated. All comparisons are made relative to untreated controls and* P-*values below <0.05 were regarded as statistically significant. 

## Results

***Cytotoxicity of neobaicalein on myelogenous leukemia cells***

The cytotoxic potential of 6.25-100 µM of neobaicalein was evaluated on HL-60 and K562 cells using an MTS assay. The results confirmed that, neobaicalein significantly decreased cell viability in a dose-dependent manner in HL-60 (at 25, 50, and 100 µM) and K562 (at 50 and 100 µM) cells (*P*<0.005). According to our results, neobaicalein half maximal inhibitory concentration (IC_50_) values in the survival curve on HL-60 and K562 cells are equal to 40.5 and 84.8 µM, respectively (Figure 1a & b). 

***Comparative analysis of cell growth inhibition by neobaicalein, wogonin, 6-hydroxyflavone, and baicalein***

In order to evaluate and compare the optimum cytotoxic concentration (CC) of isolated flavonoids with 6-hydroxyflavone and baicalein that causes a 50% cytotoxic effect (CC_50_) against malignant cells cytotoxicity, another MTS assay was carried out with different concentrations (6.5-100 µM) on HL-60 and K562 cells. All the compounds showed cytotoxic effects on the HL-60 and K562 cells in a dose-dependent manner and among them, neobaicalein was found to be the most effective compound (Figure 1a & b) with toxicity started at concentrations as low as 6.5 µM. The structures of the four compounds are shown in Figure 1c.

***Flow cytometry analysis and apoptosis induction by neobaicalein in HL-60 and K562 cells***

**Figure 2:**
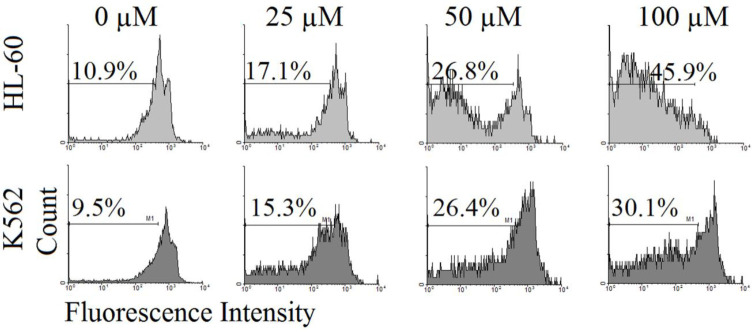
Effect of neobaicalein (25, 50, and 100 μM) on apoptosis by flow cytometry with propidium iodide (PI) in HL-60 and K562 cells. Sub-G1 peak as an indicative of apoptotic cells was induced in neobaicalein-treated cells but not in control cells

According to our results, incubation of HL-60 and K562 cells with 25, 50, and 100 µM neobaicalein for 48 hr, leads to a significant increase in the number of apoptotic cells. The percentages of apoptotic cells were 17.1%, 26.8%, and 45.9% in HL-60 cells and 15.3%, 26.4%, and 30.1% in K562 cells, however, these percentages were significantly lower in untreated cells (10.9% and 9.5% for HL-60 and K562 cells, respectively) (Figure 2). 

***Caspase activity in neobaicalein-induced apoptosis in K562 cells***

**Figure 3:**
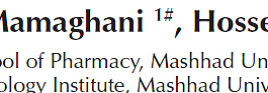
Fold activity of caspases in K562 cells after 48 hr of treatment with 25 and 50 µM of neobaicalein. Results are the mean±SEM of three independent experiments

For neobaicalein-apoptosis induction at 25 and 50 µM in K562 cells, we measured the caspase activities using synthetic pNA-conjugated substrates. Results indicated that neobaicalein induced apoptosis because when K562 cells were exposed to neobaicalein for 48 hr, the activity of caspases -3 (*P*<0.0001), -8 (*P*<0.0001), and -9 (*P*<0.01) were significantly elevated (Figure 3).

***Western blot analysis and apoptosis induction by neobaicalein in HL-60 and K562 cells***

**Figure 4:**
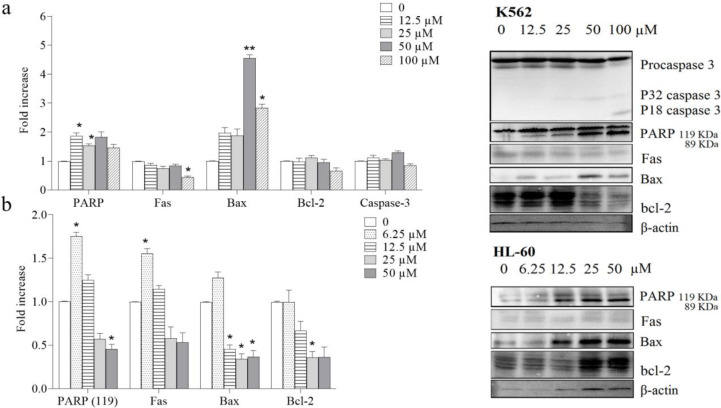
Effect of neobaicalein on protein levels of PARP, Fas, Bax, Bcl-2, and caspase 3 by western blot analysis in K562 and HL-60 cells. (a) Treatment with 12.5–100 μM of neobaicalein could significantly increase (at 12.5 and 25 µM) cleaved PARP (*P*<0.05) and (at 50 and 100 µM) Bax (*P*<0.01) in K562 cells, (b) Treatment with 6.25-50 μM of neobaicalein could significantly increase (at 6.25 µM) cleaved PARP (*P*<0.05) and Fas (*P*<0.05), and significantly decreased (at 25 µM) Bcl-2 (*P*<0.05) levels to a level near that of the related control in HL-60 cells. β-Actin was used as the loading control. Western blots shown were representative of three independent experiments. Values are the mean±SEM of three independent experiments in triplicate. *P<0.05, ** *P*<0.01, ***P<0.001 compared with the control group

In the present study, treating the HL-60 cells with 6.25 µM neobaicalein significantly increased cleaved PARP (*P*<0.05), Fas (*P*<0.05), and with 25 µM significantly decreased Bcl-2 (*P*< 0.05) levels to a level near that of the related control. Also, the results showed that neobaicalein induced significant apoptosis in K562 via increasing (at 12.5 and 25 µM) cleaved PARP (*P*<0.05) and (at 50 and 100 µM) Bax (*P*<0.01) levels to a level near that of the related control (Figure 4).

**Table 1:** ^1^H (500 MHz), ^13^C NMR (125 MHz), and HMBC spectral data of neobaicalein in CDCl_3_

Position	Neobaicalein		
	^13^C-NMR δ_C_(ppm)	^1^H-NMR δ_H_(ppm),* J*(Hz)	HMBC (H→C)
1		-	
2	162	-	
3	112.2	6.62 s	C-10, C-2
4	183.3	-	
5	152.9	-	
6	136.3	-	
7	146.4	-	
8	132.8	-	
9	149.7	-	
10	106.8	-	
1'	108.7	-	
2'	158.8	-	
3'	102.9	6.66 d (*J*=8.5 Hz)	C-1'
4'	132.8	7.28 t (*J*=8.5 Hz)	C-6'
5'	110.1	6.51 d (*J*=8.5 Hz)	C-1'
6'	156.4	-	
6'-OCH_3_	55.8	3.80 s	C-2'
6-OCH_3_	61.1	3.92 s	C-6
7- OCH_3_	61.2	4.10 s	C-5
8- OCH_3_	62.1	3.91 s	C-8
2'-OH	-	8.23 s	
5-OH	-	12.23 s	C-9, C-6, C-10

PARP: Poly (ADP-ribose) polymerase; Bax: Bcl-2 associated X-protein

## Discussion

*Scutellaria *is a recognized source of flavonoids that are widely used for their therapeutic properties in the treatment of cancers (28,29). *S.* *radix* has been recognized to display inhibitory effects on the viability of malignant cell lines. *S. litwinowii* extract showed significant cytotoxic effects against tumor cell lines and induced apoptosis by causing DNA fragmentation in our previous study (11). Among more than 35 flavonoids reported in* the Scutellaria* genus, wogonin, and neobaicalein have been identified in *S. litwinowii *followed by HPLC fractionation. Both compounds have a similar flavone backbone and inhibited tumor cell growth *in vitro*. The placement of hydroxyl and methoxy groups in the chemical structure of wogonin and neobaicalein differentiates these two phytochemicals from each other. In spite of precise mechanistic studies on flavonoids of the* Scutellaria *genus including wogonin (30,31), there are no reports on the biological activities of neobaicalein. Our previous study showed the cytotoxic activity of the compound on HeLa cells (15). Here we have compared the growth inhibition activity of neobaicalein, wogonin, 6-hydroxyflavone, and baicalein on HL-60 and K562 cells. We have also studied the role of neobaicalein in the induction of apoptotic cell death. 

 The results presented here designate that at least part of the cytotoxicity of the CH2Cl2 fraction of *S. litwinowii *to the myelogenous cell lines that we assessed can be attributed to neobaicalein and wogonin. Likewise, flavones derived from *Scutellaria* showed growth inhibitory activity against many human tumor cell lines with minimal cytotoxic effects on normal cells. Neobaicalein has been shown to be responsible as one of the most important elements in the cytotoxicity of *S.* *radix* (32). Thayer *et al.* reported that neobaicalein is one the most potent of the six flavonoids examined for cytotoxicity against L1210 cells *in vitro* with an IC_50_ value of 1.5 μg/ml (=4 μM) (10). Researchers demonstrated the inhibitory effect of neobaicalein on the growth of androgen-sensitive human prostate adenocarcinoma NCaP and human prostate cancer PC-3 cells. Both cell lines were sensitive to the cytotoxic effects of neobaicalein with IC_50_ values of 22 and 35 μM against LNCaP and PC-3 cells respectively after 72 hr (31).

Concentration-dependently neobaicalein exhibited cytotoxic activity against HL-60 and K562 cells with IC_50_ values of 40.5 and 84.8 µM, respectively after 48 hr in this study. The presence of a sub-G1 peak in the flow cytometry histogram of treated cells compared with control cells is an indicator of induction of apoptosis in growth-inhibitory effects of neobaicalein. We also determined that the cytotoxic effect of neobaicalein was also connected with the up-regulation of caspase -3, -8, and -9 key enzymes in apoptosis promotion and progression (33). The molecular mechanisms responsible for inducing apoptosis preferentially in tumor cells by wogonin, baicalein, and baicalin are largely unknown (29). Bcl-2, as one of the major antiapoptotic Bcl-2 family proteins, prevents the release of cytochrome c and triggers the caspase cascade activation, while Bax is a pore-forming pro-apoptotic protein that initiates the release of cytochrome c, prompting caspase-mediated apoptosis. Agents that lower the ratio of Bcl-2/Bax are considered attractive tools for the development of new anticancer drugs (33, 34). While a decrease in the level of Bcl-2 protein was seen after exposure of HL-60 cells to neobaicalein, an increase in the level of Bax protein occurred in K562 cells. A decrease in the Bcl-2 protein levels together with the increase in the amount of Bax, caspases -3, -8, and -9, and cleavage of PARP, confirms the role of the mitochondrial pathway in neobaicalein-induced apoptosis. Initiator caspases (cysteinyl aspartate-specific proteases) are the key substrates for the induction of programmed death through both extrinsic and intrinsic pathways of apoptosis. Once the death-inducing signaling complex (DISC) is formed, autocatalytic activation of procaspases -2, -8, or -10 promotes the extrinsic pathway, while intrinsic death signals recruit procaspase-9 to initiate the death cascade. Cleavage of the effector procaspases (caspase -3, -6, and -7) is initiated followed by the activation of the initiator caspases via both the extrinsic and intrinsic pathways of apoptosis; also they have amplification effects on caspases cascade (33). 

PARP as the first identified substrate of caspases is implicated in the cellular process of DNA repair following damages that have affected DNA function (35). PARP cleavage through apoptosis by effector caspases into p89 and p24 fragments results in suppressing PARP activity (36). Activation of caspase-8, caspase-9, and caspase-3, and cleavage of PARP is indicative of the involvement of both the extrinsic and intrinsic pathways of apoptosis in the molecular mechanisms of HL-60 and K562 cells treated with neobaicalein.

The increase of Bax following treatment of cells with neobaicalein suggests that changes in the mitochondrial membrane balance were involved in the apoptosis in HL-60 and K562 cells (Figure 4). Accordingly, the ratio of Bcl-2/Bax determines the endpoint for the cells subjected to apoptotic agents. 

Fas (CD95), a member of the tumor necrosis factor receptor family when ligated to its specific ligand induces apoptosis in cells of different origins. The absence of the Fas receptor in K562 cells is one of the examples of the differences in internal pathways of cell death in HL-60 and K562 cells that causes differences in IC_50_ values of neobaicalein against leukemia cells (37). We have studied the apoptosis induction in HL-60 and K562 cells at the time neobaicalin has shown the most cytotoxic effect in the MTS assay. Since the expression of the Fas receptor did not change in K562 cells after treatment with neobaicalein, it seems that the neobaicalein-induced apoptosis is not dependent on the Fas/Fas-L system (38). Zou *et al.* reported similar results in which adriamycin and doxorubicin showed no change in the expression of the Fas receptor in HL-60 and K562 cells.

The viability assays we carried out on HL-60 and K562 cells by neobaicalein, wogonin, 6-hydroxyflavone, and baicalein showed differences in the apoptotic potency of these four compounds (15). In a study, Tayarani-Najaran* et al.* examined the cytotoxicity of neobaicalein, wogonin, 6-hydroxyflavone, and baicalein on HeLa cells. They demonstrated that neobaicalein and wogonin exhibited significant cytotoxic effects against HeLa cell lines by inhibiting proliferation with the IC50 values of 46.62 and 79.34 µM, respectively, and inducing apoptotic cell death (15). In another study, it was observed that neobaicalein (skullcapﬂavone II) and wogonin as the active components of *Scutellaria pinnatifida* showed cytotoxic effects via decreasing viability, increasing apoptotic cell death and the amount of Bax and cleavage of PARP protein levels of K562 and HL-60 cell lines (39). It has been reported that four purified constituents baicalein, wogonin, neobaicalein, and skullcapflavone in *S. baicalensis* displayed cell growth inhibition in LNCaP cells and PC-3 cells. Baicalein, wogonin, neobaicalein, and skullcapflavone inhibited the growth of LNCaP cells with IC_50_ values 13, 42, 22, and 11 μmol/L, respectively. Also, baicalein, wogonin, and neobaicalein decreased cell viability of PC-3 cells with the IC_50_ values 25 μmol/l, 50 μmol/l, and 35 μmol/l, respectively (40).

Based on our results, neobaicalein, wogonin, 6-hydroxyflavone, and baicalein showed cytotoxic effects in a dose-dependent manner on the HL-60 and K562 cells and among them, neobaicalein was found to be the most effective compound (Figure 1a & b). Overall, our findings showed that neobaicalin causes an increment in cleaved PARP and Bax levels in K562 cells and also induces increasing cleaved PARP, Fas, apoptotic cell death, and decreasing Bcl-2 levels in HL-60 cells, it is suggested that death receptors and mitochondrial pathways are involved in neobaicalin induced apoptosis.

## Conclusion

Taken together, the present findings provide evidence for the anti-cancer potential of neobaicalein in HL-60 and K562 cells. There is also evidence that neobaicalin excreted its cytotoxicity through both death receptor and mitochondrial pathways of apoptosis and apparently, this compound is a promising agent in the future of cancer therapy.

## Authors’ Contributions

SAE, ER, SHM, NVM, JA, and HP performed the experiments, computations, analyzed the data, and wrote the manuscript. ZTN conceived, designed, and supervised the project, wrote the manuscript, and provided financial support and approved the final manuscript.

## Conflicts of Interest

The authors declare that there are no conflicts of interest. 
